# Novel *WDR72* Mutations Causing Hypomaturation Amelogenesis Imperfecta

**DOI:** 10.3390/jpm13020326

**Published:** 2023-02-14

**Authors:** Youn Jung Kim, Hong Zhang, Yejin Lee, Figen Seymen, Mine Koruyucu, Yelda Kasimoglu, James P. Simmer, Jan C.-C. Hu, Jung-Wook Kim

**Affiliations:** 1 Department of Pediatric Dentistry & DRI, School of Dentistry, Seoul National University, Seoul 03080, Republic of Korea; 2 Department of Biologic and Materials Sciences & Prosthodontics, School of Dentistry, University of Michigan, Ann Arbor, MI 48109, USA; 3 Department of Paediatric Dentistry, Faculty of Dentistry, Altinbas University, Istanbul 34147, Turkey; 4 Department of Pedodontics, Faculty of Dentistry, Istanbul University, Istanbul 34116, Turkey; 5 Department of Molecular Genetics & DRI, School of Dentistry, Seoul National University, Seoul 03080, Republic of Korea

**Keywords:** hereditary, mutation, *WDR72*, exon deletion, enamel defects

## Abstract

Amelogenesis imperfecta (AI) is a heterogeneous collection of hereditary enamel defects. The affected enamel can be classified as hypoplastic, hypomaturation, or hypocalcified in form. A better understanding of normal amelogenesis and improvements in our ability to diagnose AI through genetic testing can be realized through more complete knowledge of the genes and disease-causing variants that cause AI. In this study, mutational analysis was performed with whole exome sequencing (WES) to identify genetic etiology underlying the hypomaturation AI condition in affected families. Mutational analyses identified biallelic *WDR72* mutations in four hypomaturation AI families. Novel mutations include a homozygous deletion and insertion mutation (NM_182758.4: c.2680_2699delinsACTATAGTT, p.(Ser894Thrfs*15)), compound heterozygous mutations (paternal c.2332dupA, p.(Met778Asnfs*4)) and (maternal c.1287_1289del, p.(Ile430del)) and a homozygous 3694 bp deletion that includes exon 14 (NG_017034.2:g.96472_100165del). A homozygous recurrent mutation variant (c.1467_1468delAT, p.(Val491Aspfs*8)) was also identified. Current ideas on WDR72 structure and function are discussed. These cases expand the mutational spectrum of *WDR72* mutations causing hypomaturation AI and improve the possibility of genetic testing to accurately diagnose AI caused by *WDR72* defects.

## 1. Introduction

Amelogenesis imperfecta (AI) is a collection of rare genetic diseases affecting tooth enamel quantitatively and/or qualitatively [1]. In a strict definition, AI was suggested to include only non-syndromic hereditary enamel defects without any other non-oral symptoms [2]. However, accompanying symptoms have been identified in cases with *DLX3*, *FAM20A*, and *WDR72* mutations after the initial observation of enamel defects. Identifying AI causative genes and variants has advanced our understanding of the pathologic mechanism of AI. It is important to keep in mind that AI condition can be isolated affecting only the dentition, but it can be part of a syndromic condition [3,4,5,6] where early and accurate diagnosis can improve the patients’ prognosis.

Enamel defects can also arise from non-genetic factors, such as environmental or nutritional causes, resulting in phenocopy, which is sometimes difficult to differentiate from AI [7]. Furthermore, the list of AI-causing genes is expanding significantly with the application of modern genetic techniques such as whole exome and whole genome sequencing as well as long-read sequencing [8,9]. Therefore, candidate gene sequencing is no longer feasible or recommended for the identification of the AI-causing mutation(s) except for some cases with very distinct clinical phenotypes: hypoplastic enamel, gingival hyperplasia, multiple eruption failures in *FAM20A* mutations [10,11], horizontal hypoplastic grooves in *ENAM* mutations [12,13], and irregular vertical grooves caused by lyonization or X-inactivation in *AMELX* mutations [14].

The classic Witkop AI classification taking into consideration clinical phenotype and inheritance pattern distinguishes four major AI types with 14 subtypes: hypoplastic, hypocalcification, hypomaturation, and hypomaturation–hypoplastic with taurodontism [2]. Because sometimes it is difficult to distinguish hypocalcification and hypomaturation AI subtypes, a simplified classification of hypoplastic and hypomineralization has been suggested [15]. To date, six genes have been identified to cause autosomal recessive hypomaturation AI (Table 1): *MMP20* (matrix metalloproteinase 20) [16], *KLK4* (kallikrein related peptidase 4) [17], *WDR72* (WD repeat domain 72) [18], *ODAPH* (odontogenesis associated phosphoprotein, aka *C4orf26*) [19], *SLC24A4* (solute carrier family 24 member 4) [20] and *GPR68* (G protein-coupled receptor 68) [21]. The mixed enamel phenotype of hypoplastic and hypomaturation AI can be caused by heterozygous *DLX3* (distal-less homeobox 3, OMIM *600525) mutations and missense mutations in the middle part of *AMELX* (amelogenin X-linked, OMIM *300391) [3,14,22,23,24].

The *WDR72* gene has 20 exons (NM_182758.4, WD repeat-containing protein 72 isoform a), and the translation begins from exon 2 and terminates in exon 20, the last exon [25]. There are seven more mRNA transcript variants that use non-coding 5′ alternative exon(s). In addition, exon 7 was deleted in isoform XM_047432345.1. An interesting isoform (NM_001277176.2, WD repeat-containing protein 72 isoform b) has only three exons and encodes only 88 amino acids. The first exon is located between exons 18 and 19, and the last two exons share the sequences of exons 19 and 20 of NM_182758.4. Expression in normal tissues shows higher expressions in the kidney and thyroid followed by the liver and salivary glands (https://www.ncbi.nlm.nih.gov/gene/256764 (accessed on 18 January 2023)). Tissue-specific mRNA expression and potential functions await discovery.

In this study, we recruited families with a hypomaturation AI phenotype and performed mutational analysis by whole exome sequencing (WES). The mutational analyses revealed novel homozygous and compound heterozygous *WDR72* mutations in three families and a recurrent homozygous mutation in a fourth family. This report expands the mutational spectrum of the *WDR72* gene causing enamel hypomaturation and advances our understanding of normal and pathologic amelogenesis.

## 2. Materials and Methods

### 2.1. Enrollment of the Study Families

The protocols of this study and patient consents were independently reviewed and approved by the institutional review boards of Seoul National University Dental Hospital (CRI05003G and 9 December 2021), Istanbul University (no. 2008/931 and 20 September 2019), and the University of Michigan (H03-00001835-M1 and 6 May 2021). Informed consent was obtained from all participating individuals after explaining the nature of the study. Clinical examination and sample collection were performed according to the principles in the Declaration of Helsinki. Pedigrees were drawn from the family histories.

### 2.2. Genomic DNA Isolation and WES

Genomic DNA was isolated from saliva samples by a conventional method with the NucleoSpin Blood L kit (Macherey-Nagel GmbH & Co., Düren, Germany) according to the manufacturer’s instructions. After measuring their quality and quantity, DNA samples from selected individuals were submitted for WES (Yale Center for Mendelian Genomics, West Haven, CT, USA, Theragen Etex Bio Institute, Suwon-si, Gyeonggi-do, Korea, and BGI, Shenzhen, China). Following exome capture, paired-end sequencing reads were generated.

### 2.3. Bioinformatic Analysis

WES reads were processed using a series of bioinformatic analyses as previously described [26] (Table S1). Briefly, after trimming to remove the adapter sequences, the reads were aligned to the reference human genome assembly (hg38). Bioinformatic analysis programs, such as Samtools and Genome Analysis Tool Kit, were used to generate a list of sequence variants [27,28]. The dbSNP build 147 database was used for the annotation of the sequence variants with ANNOVAR [29], and the annotated variants were filtered with a minor allele frequency (MAF) of 0.01.

### 2.4. Sanger Sequencing

The identified mutations and the segregation among the family members were confirmed by Sanger sequencing. The polymerase chain reaction (PCR) primer pairs for the *WDR72* gene were described previously [18]. Sanger sequencing was performed for all participating family members at Macrogen (Seoul, Korea) or Eurofins Genomics (Louisville, KY, USA). The identified *WDR72* mutations were submitted to the ClinVar database (https://www.ncbi.nlm.nih.gov/clinvar/ (accessed on 18 January 2023), Submission ID: SUB12540273, SUB12549239, SUB12549244, and SUB12540289).

### 2.5. Exon Deletion Characterization

Two intra-intron PCRs and flanking PCR across exon 14 were performed to characterize the size and location of the deletion identified in family 4. The primer pairs for intra-intron 13 (656 bp) were: I13-F (forward) 5′-TCCTTATGTCATGTGTATGCC-3′ and I13-R (reverse) 5′-TAAAATTCACTGTCTTTCTGAGAAG-3′. The primer pairs for intra-intron 14 (711 bp) were: I14-F (forward) 5′-TGGCTGCATTATGCTCTATG-3′ and I14-R (reverse) 5′-GGTACTCTGCTGGGCTGAAA-3′. Flanking PCR was performed with the I13-F and I14-R primers (475 bp only in the mutant allele). Each amplification band was sequenced by Sanger sequencing at Eurofins Genomics.

## 3. Results

### 3.1. Family 1

**Figure 1:**
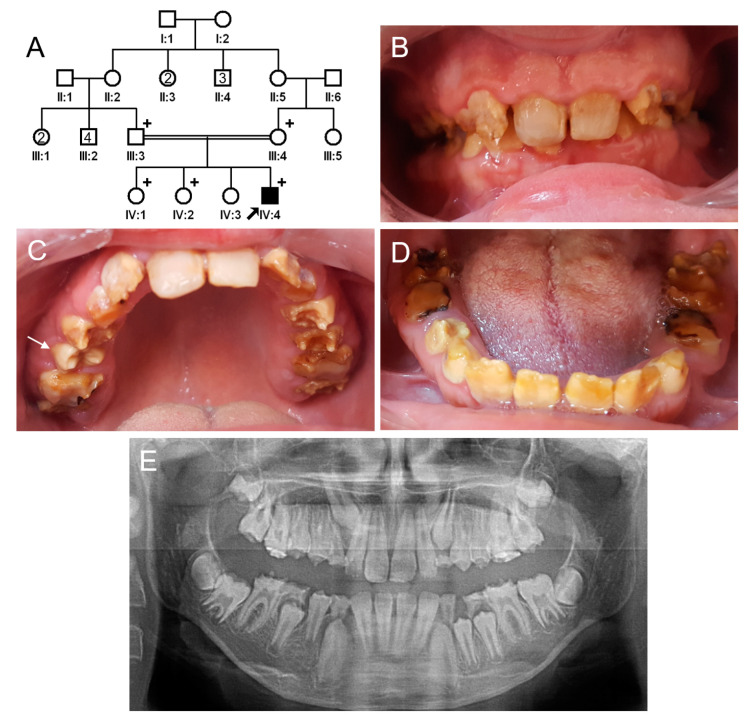
Pedigree, clinical photos, and panoramic radiograph of family 1. (**A**) Pedigree of family 1. The black symbol indicates the affected individual, and the proband is indicated by a black arrow. Plus signs above the symbols indicate participating individuals. The number inside the symbol indicates the number of siblings with the same gender. (**B**–**D**) Clinical photos of the proband at age 12 years. Maxillary central incisors have been treated with direct composite resin restorations. A newly erupted tooth (white arrow) exhibits a normal shape but discoloration before enamel fracture or attrition. Otherwise, all affected teeth show yellow-brown discoloration and enamel fractures and accelerated attritions. (**E**) Panoramic radiograph of the proband at age 12 years. Radiographically, unerupted teeth show normal crown shape with a reduced enamel radiodensity. Severe attrition and fractures can be seen in some teeth, especially in the first molars and deciduous second molars.

The proband of family 1 was a 12-year-old male, the fourth child from a consanguineous Turkish family (Figure 1). Pregnancy and delivery were uneventful, and he had no remarkable past medical history. His primary and permanent dentitions exhibited characteristic features of hypomaturation amelogenesis imperfecta: generalized yellow-brown discoloration, normal or near-normal thickness of enamel before tooth eruption, rapid dental attrition, or enamel fractures due to weak enamel. He also exhibited class II malocclusion with a deep overbite. There were no other family members with a similar dental phenotype. A recessive or de novo dominant mutation, therefore, was suspected.

**Figure 2:**
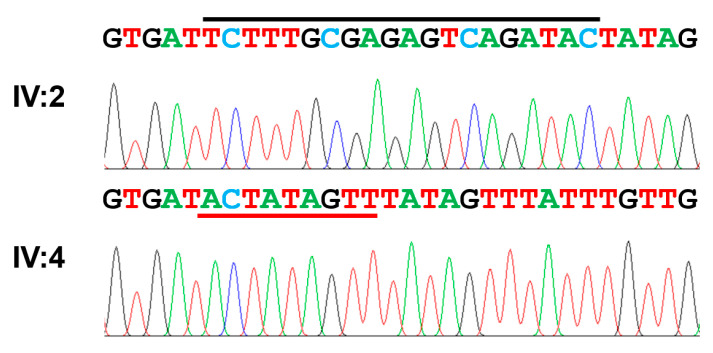
Sequencing chromatograms. Normal nucleotides (marked with a black bar above the sequence) in the upper chromatogram of the normal individual, IV:2, are deleted, and the novel nucleotides (marked with a red bar above the sequence) are inserted in the lower chromatogram of the proband, IV:4. The deletion and insertion mutation is described as c.2680_2699delinsACTATAGTT (NM_182758.4) and p.(Ser894Thrfs*15).

Investigation of the filtered list of variants obtained by WES of the proband revealed only one variant among the known AI-causing genes. The variant was a homozygous deletion mutation of 20 coding nucleotides replaced with an insertion of 9 novel nucleotides (NM_182758.4: c.2680_2699delinsACTATAGTT) in exon 15 of the *WDR72* gene (Figure 2). The deletion and insertion were predicted to cause a frameshift (NP_877435.3: p.(Ser894Thrfs*15)). This mutation was not listed in any of the mutation databases. The mutant allele with this frameshift would not produce a truncated protein because the mutant mRNA would be degraded by the nonsense-mediated decay system (NMDS) due to the premature termination codon (PTC) in the early exon (*WDR72* has 20 exons and translation begins in exon 2 and terminates in exon 20).

### 3.2. Family 2

**Figure 3:**
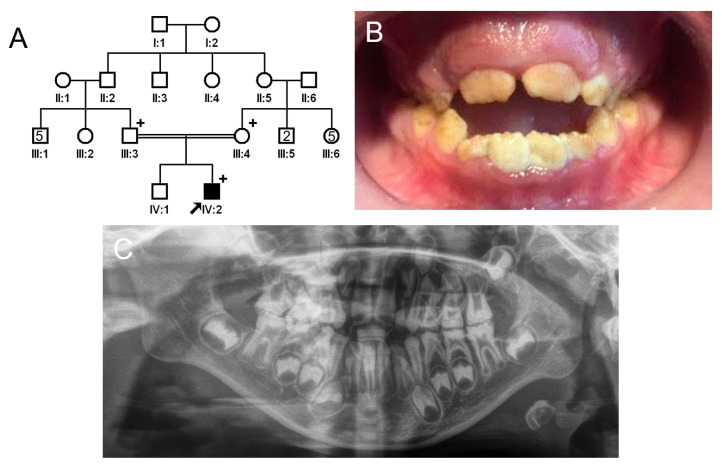
Pedigree, clinical photo, and panoramic radiograph of family 2. (**A**) Pedigree of family 2. The black symbol indicates the affected individual, and the proband is indicated by a black arrow. A plus sign above the symbol indicates participating individuals. (**B**) Clinical photo of the proband at age 7 years. He has an anterior open bite with a constricted maxilla. (**C**) Panoramic radiograph of the proband at age 7 shows enamel fractures and attrition in almost all posterior teeth including first molars. The affected enamel has reduced radiopacity, making it similar to the underlying dentin.

The proband of family 2 was a 7-year-old male, the second child from a consanguineous Turkish family (Figure 3). He had no other remarkable past medical history. His newly erupted permanent anterior teeth exhibited brown discoloration but a normal shape because the hypomineralized enamel had not yet fractured. However, the remaining deciduous teeth and first permanent molars exhibited apparent attrition and enamel fractures. There were no other affected family members except for the proband; therefore, a recessive or de novo mutation was suspected.

**Figure 4:**
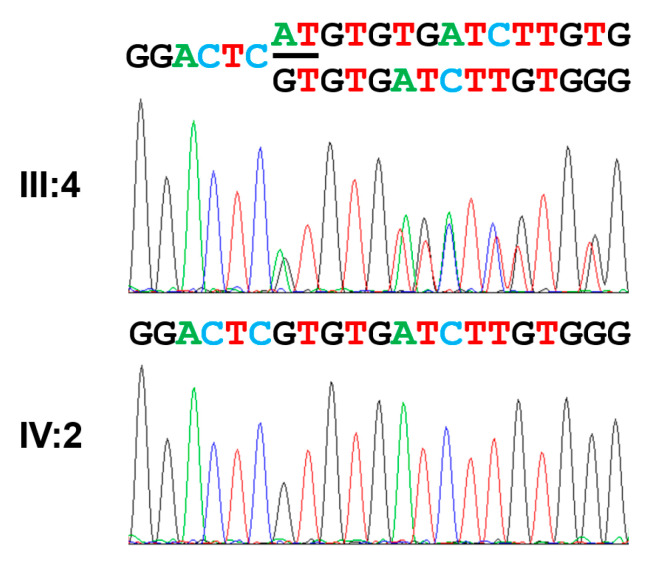
Sequencing chromatograms. Heterozygous deletion of AT (marked with a black bar under the sequence) in the normal but heterozygous individual, III:4, results in a mixed peak from the deletion. A sequencing chromatogram of the proband (IV:2) shows a homozygous AT nucleotides deletion. The deletion mutation is indicated as c.1467_1468delAT (NM_182758.4) and p.(Val491Aspfs*8).

There were two variants in the known AI-causing genes among the filtered list of annotated variants: a homozygous missense variant (c.3025G>A, p.(Val1009Ile)) in exon 18 of the *WDR72* gene and a homozygous deletion variant (c.1467_1468delAT, p.(Val491Aspfs*8)) in exon 12 of the *WDR72* gene (Figure 4). The missense variant is listed in the dbSNP (rs371227564) and GnomAD v2.1.1 (https://gnomad.broadinstitute.org/ (accessed on 18 January 2023)). Even though it is rare (15/282526 in GnomAD), the in silico PolyPhen2 prediction was benign, and the CADD score was very low (0.042). Most importantly, the existence of an upstream frameshift mutation would remove the disease-causing potential of this variant. The AT deletion in exon 12 was independently reported several times in the database (rs606231462) and therefore suggested to be a mutational hot spot [25,30,31]. A previous report identified two different mutant alleles (Mexican and Turkish families) with the same mutation. Haplotype analysis revealed that the mutant allele in this family was identical to the previously reported one from a Turkish family, identical by descent. This frameshift mutation would result in PTC, and the mutant mRNA would be degraded by the NMDS as well.

### 3.3. Family 3

**Figure 5:**
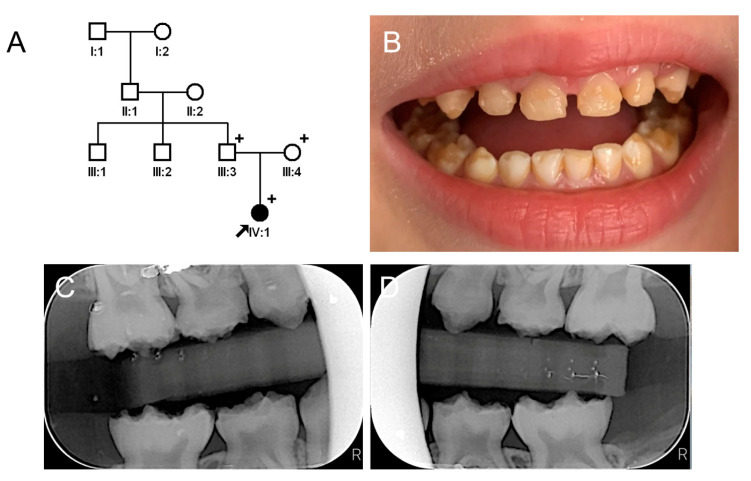
Pedigree, clinical photo, and bitewing radiographs of family 3. (**A**) Pedigree of family 3. The black symbol indicates the affected individual, and the proband is indicated by a black arrow. A plus sign above the symbol indicates participating individuals. (**B**) Clinical photo of the proband at age 4 years. She has a dull yellowish-brown discoloration of her dentition, and the affected enamel shows attrition and fractures. (**C**,**D**) Bitewing radiographs of the proband at age 4 years show enamel fractures with reduced radiopacity.

The proband of family 3 was a 4-year-old female from a nonconsanguineous Caucasian family (Figure 5). She had no other remarkable past medical or dental history except for a generalized yellowish-brown discolored deciduous dentition. Her teeth showed attrition and enamel fractures mostly in the cuspal area. The remaining enamel barely contrasted with the underlying dentin, suggesting a reduced degree of mineralization. She was the only affected individual among her family members; therefore, a spontaneous or recessive mutation was suspected.

**Figure 6:**
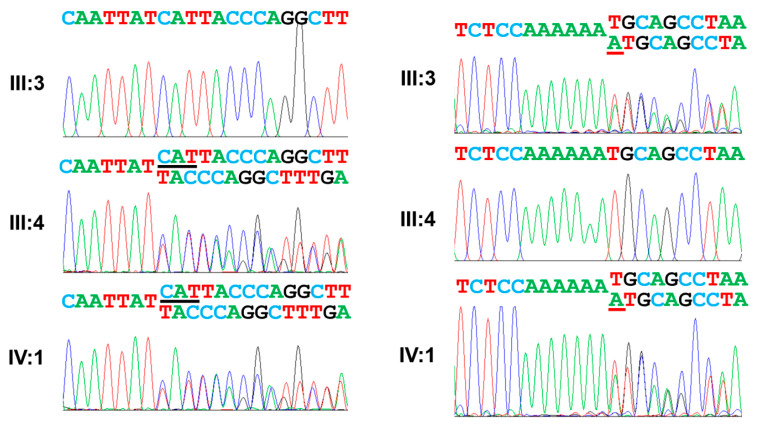
Sequencing chromatograms. The left side shows the maternal mutation (c.1287_1289del, p.(Ile430del)). Deleted nucleotides are indicated by black underline. The right side shows the paternal mutation (c.2332dupA, p.(Met778Asnfs*4)) with the duplicated nucleotide indicated by a red underline.

Trio analysis of the WES revealed compound heterozygous mutations in the *WDR72* gene: a single nucleotide duplication (c.2332dupA) causing a frameshift (p.(Met778Asnfs*4)) in exon 15 in the paternal allele and a non-frameshift three nucleotide deletion (c.1287_1289del) resulting in a single codon deletion (p.(Ile430del)) in exon 11 in the maternal allele (Figure 6). The frameshift mutation in the paternal allele was listed in dbSNP (rs764406738) and GnomAD v2.1.1 (3/251086), and the mutant transcript would be degraded by the NMDS. The maternal mutation was not listed in any of the databases and would delete an amino acid at codon position 430, which is in the 5th WD repeat domain among the seven predicted WD repeats in WDR72. This deletion would result in structural instability, causing protein degradation by the misfolding surveillance system or conformational changes damaging normal protein function.

### 3.4. Family 4

**Figure 7:**
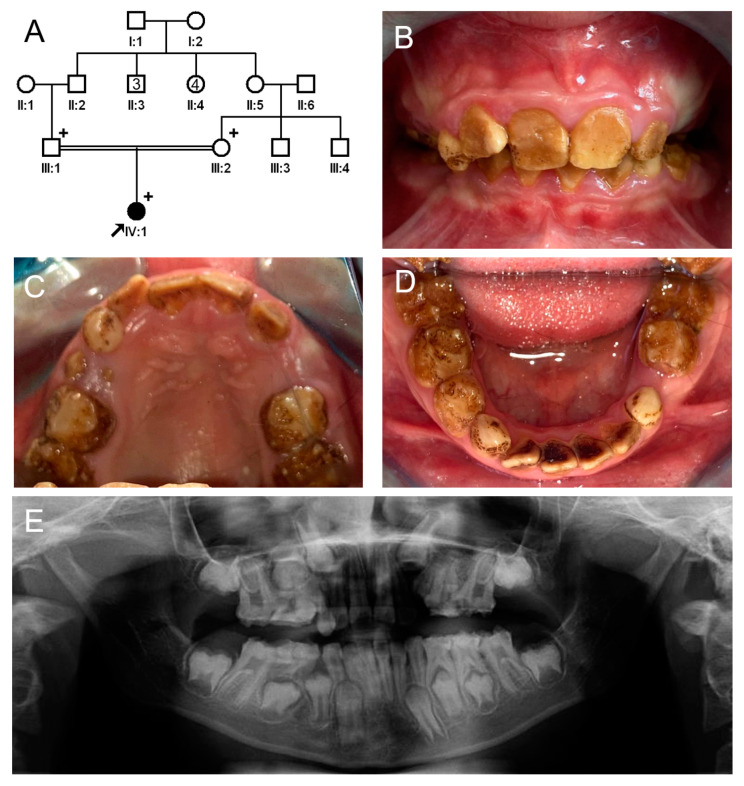
Pedigree, clinical photos, and panoramic radiograph of family 4. (**A**) Pedigree of family 4. The black symbol indicates the affected individual, and the proband is indicated by a black arrow. A plus sign above the symbol indicates participating individuals. The number inside the symbol indicates the number of siblings with the same gender. (**B**–**D**) Clinical photos of the proband at age 10 years. Permanent and deciduous dentitions show dark brown discoloration and exhibit enamel fractures and accelerated attrition. Black external stain is observed on most teeth. (**E**) A panoramic radiograph of the proband at age 10 years shows irregular crown surfaces and enamel with reduced radiopacity and fractures. However, the developing crowns are normal in shape.

The proband of family 4 was a 10-year-old female from a consanguineous Turkish family (Figure 7). She had no remarkable past medical history. Her deciduous and permanent dentition had a dark brown discoloration and exhibited enamel breakdown and accelerated attritions. There were black stains throughout the dentition. She had no kidney symptoms, and the pediatric nephrology department confirmed that she had no acidosis, and her renal and tubule functions were normal. She was the only affected individual in her family; therefore, a recessive or de novo mutation was suspected.

**Figure 8:**
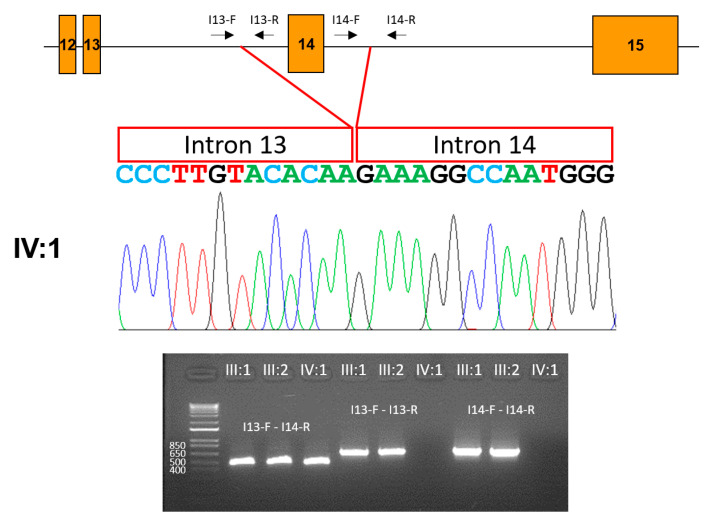
Characterization of the deletion. A partial gene diagram is shown at the top. Exons are indicated with orange boxes, and exon numbers are in the boxes. Introns are shown as horizontal lines. Locations and directions of the primer binding sites are shown above the introns. Deletion points are indicated with red lines. The sequencing chromatogram of the proband is shown in the middle. The intron 13 and 14 sequences are shown as boxes above the nucleotide sequences. The agarose gel image is shown at the bottom. PCR reaction with I13-F and I14-R results in a 475 bp band from the mutant allele but a 4169 bp band from the wild-type allele. PCR amplifications within intron 13 (I13-F and I13-R, 656 bp) and 14 (I14-F and I14-R, 711 bp) did not produce PCR bands from the proband.

Mutational analysis of the trio data of WES failed to identify a disease-causing variant. Then, checking the low coverage regions in the proband’s known AI-causing genes suggested a deletion in the *WDR72* gene. The deleted region included exon 14 and was characterized by PCR reactions (Figure 8). The length of the genomic deletion was 3694 bp (NG_017034.2:g.96472_100165del) and would change the cDNA sequence by the deletion of exon 14 (c.1766_1964del), causing a frameshift (p.(Gly589Valfs*16)). The parents were heterozygous, and the proband was homozygous for this deletion. The mutant transcript would be degraded by the NMDS due to PTC.

## 4. Discussion

The tryptophan-aspartate (WD) or WD40 repeat sequences are characterized by a conserved sequence of up to 40 to 60 nucleotides with WD at the C terminus and glycine-histidine (GH) residues at 11–24 amino acids from the N terminus [32]. However, the sequence repeat contains no absolutely conserved amino acids. These repeats are tandemly repeated 4 to 16 times typically and encode the blades of a β propeller protein structure. WD repeat-containing proteins fold into a seven-bladed β propeller with each blade of a four-stranded anti-parallel β sheet typically [33]. However, the components in the repeats, such as GH and WD dipeptides, and the length of the repeats are not conserved absolutely; therefore, their inclusions in predictions are sometimes missed. There could be at least one additional non-WD domain or hidden repeat in the protein structure [34]. 

Earlier reports described seven WD repeats in the WDR72 protein with computer prediction tools such as REP (homology-based REPeat finding method) [35] and SMART (Simple Modular Architecture Research Tool, https://smart.embl.de/smart/show_motifs.pl?ID=Q3MJ13, (accessed on 18 January 2023)) [36]. However, it was also predicted later as 8 or 11 WD40 repeats (NM_182758.2 and NP_877435.3 in https://www.ncbi.nlm.nih.gov/, (accessed on 18 January 2023)). Furthermore, a recent publication used molecular modeling with the known crystal structure of yeast β′-COP (coat protein), an essential subunit in the eukaryotic COPI vesicle coat assemblies (https://www.rcsb.org/structure/3mkq, (accessed on 18 January 2023)), which suggested two seven-bladed β propeller structures [37]. Further study on the structure of the WDR72 protein would clarify these discrepancies and uncertainty.

The β propeller structure provides a platform for protein interaction and assembly, and the WD repeat proteins are involved in many cellular functions, such as signal transduction, intracellular transport, and cytoskeletal organization [33]. Autosomal recessive hypomaturation AI caused by *WDR72* mutations was considered an isolated form. However, its high expression in the kidney and genetic linkage of kidney function to the *WDR72* gene suggested possible impact on kidney function [38,39]. Recessive *WDR72* mutations were identified in distal renal tubular acidosis families [40], and serum acidosis was also confirmed in some AI patients with *WDR72* mutations [31] (Table 2). A kidney function test revealed that the proband in family 4 did not have acidosis, and her renal and tubule functions were normal. The kidney problem may not present in every AI case with *WDR72* mutations, and it may have a variable onset and phenotype depending on the mutations and other genetic factors as well [41]. Further genetic studies and a large-scale long-term multicenter follow-up study would be necessary to determine the genotype–phenotype relationship for cohorts with *WDR72* mutations. It is highly recommended to refer AI patients with *WDR72* mutations for periodic kidney evaluation.

It has been speculated and demonstrated that WDR72 is involved in vesicle trafficking and regulation of endocytosis [18,25,46]. Recent findings suggested a role for WDR72 in vesicular Golgi transport [47] and unexpectedly in the enhancement of the stemness of lung cancer cells [48]. However, the exact functional role of WDR72 is not yet clear. Two knockout mouse studies demonstrated that WDR72 is necessary for proper enamel formation, especially in maturation processes, and the AI phenotype is caused by a complete lack of functional WDR72, not by a dominant negative effect [37,49]. It has been shown that the SLC24A4 is mislocalized in maturation ameloblasts in the *Wdr72* null mouse [49]. SLC24A4 mediates sodium, potassium, and calcium transport. In the absence of proper calcium concentration, enamel mineralization would be disturbed. When WDR72 was linked to distal renal tubular acidosis, it helped localize a different transporter in the kidney, which is thought to be a V-type proton ATPase [42]. Therefore, WDR72 may function as a scaffold protein that likely binds to multiple proteins and localizes them together. Further investigations into the functional role and pathogenesis of *WDR72* mutations are warranted.

## 5. Conclusions

In this study, we recruited hypomaturation AI families and performed WES of selected family members. Novel homozygous and compound heterozygous *WDR72* mutations in three families and a recurrent homozygous mutation in one family were identified. This report expands the mutational spectrum of the *WDR72* gene and improves the possibility of using genetic testing to accurately diagnose AI caused by *WDR72* mutations.

## Figures and Tables

**Table 1:** Genes causing autosomal recessive hypomaturation AI.

Name	Chromosomal Location	Number of Exon	Length of Protein (aa)	OMIM
*MMP20*	11q22.2	10 (NM_004771.4)	483 (NP_004762.2)	*604629
*KLK4*	19q13.41	6 (NM_004917.5)	254 (NP_004908.4)	*603767
*WDR72*	15q21.3	20 (NM_182758.4)	1102 (NP_877435.3)	*613214
*ODAPH*	4q21.1	2 (NM_178497.5)	130 (NP_848592.2)	*614829
*SLC24A4*	14q32.12	17 (NM_153646.4)	622 (NP_705932.2)	*609840
*GPR68*	14q32.11	2 (NM_001177676.2)	365 (NP_001171147.1)	*601404

**Table 2:** Disease-causing mutations in *WDR72* gene.

Location	cDNA	Protein	Mode of Inheritance	References
Exon 2	c.88C>T	p.(Arg30*)	Homozygous	Khandelwal et al. (2021) [41]
Exon 3	c.154_1765del (exon 3–13 del)	p.(Ile52Alafs*25)	Homozygous	Zhang et al. (2019) [31]
Exon 5	c.477_485dup	p.(Ile159_Cys161dup)	Homozygous	Jobst-Schwan et al. (2020) [42]
Exon 5	c.377G>A	p.(Trp126*)	Homozygous	Zhang et al. (2019) [31]
Exon 8	c.764_768delGGCAG	p.(Gly255Valfs*40)	Homozygous	Jobst-Schwan et al. (2020) [42]
Exon 8	c.806_810delGGCAG	p.(Gly255Valfs*294)	Homozygous	Katsura et al. (2014) [37]
Exon 10	c.997A>T	p.(Lys333*)	Homozygous	Kuechler et al. (2012) [43]
Exon 11	c.1265G>T	p.(Gly422Val)	Homozygous	Zhang et al. (2019) [31]
Exon 11	c.1287_1289delCAT	p.(Ile430del)	Maternal	This report
Exon 12	c.1467_1468delAT	p.(Val491Aspfs*8)	Homozygous	Lee et al. (2010) [25] Wright et al. (2011) [30] Zhang et al. (2019) [31] This report
Exon 12	c.1481G>A	p.(Trp494*)	Homozygous	Khandelwal et al. (2021) [41]
Exon 13	c.1570_3148del (exon 13–18 del)	p.(Arg525Glnfs*43)	Homozygous	Hentschel et al. (2016) [44]
Exon 14	c.1766_1964del (exon 14 del)	p.(Gly589Valfs*16)	Homozygous	This report
Exon 14	c.1777A>G	p.(Arg593Gly)	Paternal	Rungroj et al. (2018) [40]
Exon 14	c.1801C>T	p.(Arg601*)	Paternal	Zhang et al. (2019) [31]
Exon 15	c.2332dupA	p.(Met778Asnfs*4)	Paternal	This report
Exon 15	c.2348C>G	p.(Ser783*)	Homozygous	El-Sayed et al. (2009) [18]
Exon 15	c.2350A>T	p.(Arg784*)	Maternal	Zhang et al. (2019) [31]
Exon 15	c.2522T>A	p.(Leu841Gln)	Maternal	Rungroj et al. (2018) [40]
Exon 15	c.2680_2699delinsACTATAGTT	p.(Ser894Thrfs*15)	Homozygous	This report
Exon 15	c.2686C>T	p.(Arg896*)	Homozygous	El-Sayed et al. (2011) [45] Rungroj et al. (2018) [40]
Exon 16	c.2857delA	p.(Ser953Valfs*20)	Homozygous	El-Sayed et al. (2009) [18]
Exon 16	c.2866G>A	p.(Arg956*)	Homozygous	Khandelwal et al. (2021) [41]
Exon 17	c.2934G>A	p.(Trp978*)	Homozygous	El-Sayed et al. (2009) [18]

* Sequences based on the reference sequence for mRNA (NM_182758.4) and protein (NP_877435.3), where the A of the ATG translation initiation codon is nucleotide 1.

## Data Availability

The data presented in this study are openly available in ClinVar (http://www.ncbi.nlm.nih.gov/clinvar (accessed on 20 January 2023)), submission ID: SUB12540273, SUB12549239, SUB12549244, and SUB12540289.

## Supplementary Materials

The following is available online at https://www.mdpi.com/article/10.3390/jpm13020326/s1, Table S1: Statistics for exome sequencing.
