# Supplementary material for: Novel WDR72 Mutations Causing Hypomaturation Amelogenesis Imperfecta
Source: J Pers Med. 2023 Feb 14;13(2):326. doi: 10.3390/jpm13020326 (PMC9965932; doi:10.3390/jpm13020326)
Supplement: Supplementary file 1 [file jpm-13-00326-s001.zip › jpm-2204323-supplementary.pdf]

Novel *WDR72* Mutations Causing Hypomaturation Amelogenesis Imperfecta

Youn Jung Kim[1], Hong Zhang[2], Yejin Lee[1], Figen Seymen[3], Mine Koruyucu[4], Yelda Kasimoglu[4], James P. Simmer[2], Jan C.-C. Hu[2], Jung-Wook Kim[1,5*]

[1]Department of Pediatric Dentistry & Dental Research Institute
School of Dentistry, Seoul National University, Seoul, Korea.
[2]Department of Biologic and Materials Sciences, School of Dentistry
University of Michigan, Ann Arbor, MI, USA.
[3]Department of Paediatric Dentistry, Faculty of Dentistry
Altinbas University, Istanbul, Turkey.
[4]Department of Paediatric Dentistry, Faculty of Dentistry
Istanbul University, Istanbul, Turkey.
[5]Department of Molecular Genetics & Dental Research Institute
School of Dentistry, Seoul National University, Seoul, Korea.

***Correspondence to:**

Jung-Wook Kim, D.D.S., M.S.D, Ph.D.
Department of Molecular Genetics
Department of Pediatric Dentistry & Dental Research Institute
School of Dentistry, Seoul National University
101 Daehak-ro, Jongno-gu, Seoul, 03080, Korea
Tel: +82-2-2072-2639, Fax: +82-2-744-3599
E-mail: pedoman@snu.ac.kr

Table S1. Statistics for exome sequencing.

Sample		Total reads	Mapping rate (%)	Median target coverage	Coverage of target region (%)	Fraction of target covered with at least	
						20X	10X
Family 1	IV:4	72,646,317	98.2	70	96.4	91.7	95.0
Family 2	IV:2	121,948,763	99.1	91	96.2	93.2	95.1
Family 3	III:3	62,823,866	97.7	59	98.7	93.7	97.7
	III:4	70,252,242	97.7	63	98.6	96.6	98.1
	IV:1	45,215,908	98.1	39	98.6	87.6	97.2
Family 4	III:1	101,299,742	98.7	91	98.8	97.8	98.3
	III:2	78,607,702	98.8	73	98.7	97.0	97.9
	IV:1	95,540,376	99.2	89	98.7	97.6	98.1
